# The Role of Pyrazolo[3,4-d]pyrimidine-Based Kinase Inhibitors in The Attenuation of CCl_4_-Induced Liver Fibrosis in Rats

**DOI:** 10.3390/antiox12030637

**Published:** 2023-03-03

**Authors:** Diana K. Ghobrial, Nefertiti El-Nikhely, Eman Sheta, Hanan M. Ragab, Sherif A. F. Rostom, Hesham Saeed, Ahmed Wahid

**Affiliations:** 1 Department of Biotechnology, Institute of Graduate Studies and Research, Alexandria University, Alexandria 21526, Egypt; 2 Department of Pathology, Faculty of Medicine, Alexandria University, Alexandria 21321, Egypt; 3 Department of Pharmaceutical Chemistry, Faculty of Pharmacy, Alexandria University, Alexandria 21500, Egypt; 4 Department of Pharmaceutical Biochemistry, Faculty of Pharmacy, Alexandria University, Alexandria 21500, Egypt

**Keywords:** liver fibrosis, celecoxib, kinase inhibitor, pyrazolo[3,4-d]pyrimidine, TGF-β

## Abstract

Liver Fibrosis can be life-threatening if left untreated as it may lead to serious, incurable complications. The common therapeutic approach is to reverse the fibrosis while the intervention is still applicable. Celecoxib was shown to exhibit some antifibrotic properties in the induced fibrotic liver in rats. The present study aimed to investigate the possible antifibrotic properties in CCl_4_-induced liver fibrosis in male Sprague–Dawley rats compared to celecoxib of three novel methoxylated pyrazolo[3,4-d]pyrimidines. The three newly synthesized compounds were proved to be safe candidates. They showed a therapeutic effect against severe CCl_4_-induced fibrosis but at different degrees. The three compounds were able to partially reverse hepatic architectural distortion and reduce the fibrotic severity by showing antioxidant properties reducing MDA with increasing GSH and SOD levels, remodeling the extracellular matrix proteins and liver enzymes balance, and reducing the level of proinflammatory (TNF-α and IL-6) and profibrogenic (TGF-β) cytokines. The results revealed that the dimethoxy-analog exhibited the greatest activity in all the previously mentioned parameters compared to celecoxib and the other two analogs which could be attributed to the different methoxylation patterns of the derivatives. Collectively, the dimethoxy-derivative could be considered a safe promising antifibrotic candidate.

## 1. Introduction

Liver fibrosis results from the liver response to injury by excessive production of extracellular matrix proteins (ECM) including collagen followed by decreased remodeling. Following many instances of injury repair, the hepatocytes can no longer repair themselves. The excessive non-remodeled proteins form scar tissue or fibrosis that if left untreated can develop portal hypertension, liver cirrhosis, and eventually hepatocellular carcinoma (HCC). In advanced cases, patients may require liver transplantation [1].

Various events are essential for the pathogenesis of liver fibrosis and its resolution. Hepatic stellate cell (HSC) activation must be avoided as this activation is a key trigger of liver fibrogenic cells (myofibroblasts) which in turn produce ECM in the liver [2]. Oxidative stress mainly by reactive oxygen species (ROS) has been implicated in HSC activation and consequent collagen synthesis [3,4]. ECM remodeling is a critical factor for liver homeostasis during hepatic injury [2]. Matrix metalloproteinases (MMPs) are involved in the breakdown of ECM in normal physiological processes [5]. Hence, a correct balance is required between MMPs and their tissue inhibitors of matrix metalloproteinases (TIMPs) to maintain the correct amount of ECM and thus homeostasis. Additionally, TIMP-1 exerts anti-apoptotic impacts on HSCs, so it triggers fibrogenesis by advancing the survival of fibrogenic cells. It is worth noting that gelatinases such as MMP-9 are also considered potential targets for the treatment of liver fibrosis and hepatic repair as they scavenge TIMP-1 and are responsible for collagen degradation. It was also found that the progression of liver fibrosis to HCC decreased with increased expression of MMP-9 [6,7,8]. Cell function is indirectly affected as well by the ECM release of inflammatory cytokines such as transforming growth factor β (TGF-β) and tumor necrosis factor-α (TNF-α) [2]. TGF-β is a main profibrogenic cytokine and, therefore, a potential target to manage fibrosis [9]. Targeting the TNF-α signaling pathway is considered a new therapeutic approach for liver fibrosis as TNF-α boosts HSC survival and hepatocyte death and triggers the immune response [10].

Although historically, liver fibrosis was believed to be irreversible, recent evidence showed that even advanced fibrosis is reversible. Concealment of the causative agent has shown effectual productivity in treating liver fibrosis of different etiologies. Currently, specific, effective, and safe antifibrotic therapies are the focus of numerous research studies [11,12]. Interestingly, celecoxib, a nonsteroidal anti-inflammatory drug (NSAID) that decreases the mediators of inflammation, and its derivatives have been developed to test their antifibrotic properties [13]. Celecoxib was reported to have an antifibrotic effect by stimulating MMP-2 activity, decreasing the expression of (TGF-β), and restoring the redox equilibrium through restoring the lipid peroxidation and glutathione levels [14,15]. In addition, celecoxib was found to reactivate apoptosis by inhibiting serine/threonine-specific protein kinase (AKT)-induced-apoptosis inhibition on HSCs and, therefore, the suppression of liver fibrosis [16].

Celecoxib analogs, i.e., synthesized compounds derived from the pyrazole nucleus (the main pharmacophore of celecoxib) were developed to enhance the COX-2 inhibitory affinity, efficacy, and selectivity, as well as to reduce possible side effects. Furthermore, some pyrazole-based compounds have been reported to have antifibrotic potentials [17,18,19]. Among these, some new pyrazolo[3,4-d]pyrimidines were reported to suppress COX-2 expression at low concentrations [20], while others possessed antineoplastic activity and were reported as kinase inhibitors [21]. Consequently, such traits of new pyrazole-based compounds would serve as potential therapeutic candidates targeting HSCs to ameliorate liver fibrosis without being toxic to normal cells. To verify this hypothesis, we first considered toxicity studies to confirm the safety of derivatives, then their fibrolytic potential was proved by their impact on the profibrogenic, proinflammatory cytokines TGF-β, IL-6, and TNFα, their role on the ECM, MMP9, and TIMP-1 enzymes, as well as their effect on oxidative stress markers.

## 2. Materials and Methods

### 2.1. Chemicals and Chemistry

This study used carbon tetrachloride (CCl_4_, Loba, Mumbai, India), carboxy methyl cellulose (CMC, Adwic, Abu-Zaabal, Egypt), celecoxib (Clx, Borg Pharma, Alexandria, Egypt), which was a generous gift from Borg Pharma, thiobarbituric acid (TBA, Loba, India), thioacetic acid (TCA, Sd Fine-Chem Limited–SDFCL, Mumbai, India), perchloric acid (HClO_4_, Central Drug House (P) Ltd.–CDH, New Delhi, India), 1,1,3,3-Tetraethoxypropane (Malonaldehyde bis (diethyl acetal), Sigma-Aldrich, Burlington, MA, USA), 50x Tris-acetate-EDTA (50x TAE, iNtRON Biotechnology, Seongnam-Si, Korea), formaldehyde (El NASR pharmaceutical chemicals Co., Cairo, Egypt), phosphate-buffered saline (PBS, Lonza, Allendale, NJ, USA), Agarose (Piochem, Giza, Egypt), 2-Mercaptorthanol (Loba, India), Triton X-100 (Loba, India), and Trizma base (Sigma-Aldrich, USA).

The target compounds were synthesized according to previously reported procedures [21].

### 2.2. In Vitro COX-1/COX-2 Inhibition Assay

The newly synthesized compounds were assayed to determine their ability to inhibit cyclooxygenases (COX-1 and COX-2) with enzyme immunoassay using Cayman colorimetric COX (ovine) inhibitor screening assay kit (Cayman Chemicals, Ann Arbor, MI, USA; Catalog No. 560131), using indomethacin and celecoxib as reference standards.

### 2.3. Cytotoxicity MTT Assay

MTT assay [22] was used to estimate cell viability potential for 24 h using hepatocellular carcinoma cell line (HepG2) as previously reported [23]. Calculation of the relative cell viability percentage was performed following the equation: % Relative cell viability = (Absorbance of treated samples/Absorbance of untreated sample) × 100.

### 2.4. Animals Experimental Design

Ninety-eight healthy adult Sprague–Dawley male rats [24,25] with an average weight of 150 ± 20 g were obtained from the animal facility at the Institute of Graduate Studies and Research, Alexandria University, Egypt. Animals were provided with a balanced commercial diet ad libitum and adapted for two weeks prior to the experiment. The Sprague–Dawley rats were randomly divided into different groups and received different treatments, as shown in Table 1. All animals were sacrificed under IP thiopental anesthesia (50 mg/kg) 48 h after the last treatment [26].

### 2.5. Biochemical Tests

Blood samples were collected using cardiac puncture in Li-heparin tubes and centrifuged at 2000× *g* for 10 min to obtain the plasma [29]. Then, the determination of hepatic biomarkers and renal profile was performed according to kits purchased from Roche Diagnostics, USA, using a Roche/Hitachi analyzer (cobas c 311, cobas c 501/502).

### 2.6. Liver Tissue Specimens

Rats were sacrificed by exsanguination following blood sample collection. The livers were excised, rinsed with cold saline, and dried on filter paper. A portion of each excised liver was put in 10% formalin solution, and then embedded in paraffin and sliced into 4–5 µm sections for histological evaluation. Another part of each liver was snap frozen and stored at −80 °C for later analyses of markers. The remaining tissue was stored in RNAlater solution (Qiagen, Germany, Mat. No. 1018087, lot no. 148052467) for qRT-PCR.

### 2.7. Histopathological and Immunohistochemistry

Sections from formalin-fixed paraffin-embedded (FFPE) liver tissues were stained with hematoxylin and eosin and Masson trichrome stain to evaluate liver necrosis and fibrosis. The scores of liver fibrosis degree were evaluated according to a 4-tiered scoring system following the criteria: score 0 = absent fibrosis where scanty fibrous tissue is seen only in portal tracts, score 1 = minimal fibrosis (expanded portal tracts with short fibrous septea may be extending from portal tracts), score 2 = mild fibrosis (long fibrous septea is extending from portal tracts with occasional bridging to other portal tracts or to central veins), score 3 = moderate fibrosis (frequent portal to portal or to central vein bridging with infrequent pseudolobule formation), and score 4 = severe fibrosis (nodular liver with evident pseudolobule formation) [30]. For collagen morphological quantification, multiple random pictures of the trichrome-stained section were taken using a microscope-adopted camera at ×400 power. The collagen-positive area stained in blue was evaluated using ImageJ software and then calculated as a percentage of the total field area. At least eight fields were assessed and the mean value was then calculated [31].

Four-micron sections were cut and mounted on positively charged slides. They were stained with smooth muscle actin (SMA) monoclonal primary antibody (Dako, Denmark; CodeIR611, lot no. 41247585) using Dako auto Stainer (autostainer Link 48). A positive control (leiomyoma) was used in each run. Immune-stained slides were assessed, and ten high power fields of each section were photographed using a Leica camera. Photos were analyzed using ImageJ software to record the immune-positive area (seen as brown staining) in each photo. Then, the positively stained area percentage was calculated as a mean of the 10 examined photos for each rat.

### 2.8. Protein Determination

Protein content determination was performed according to Bradford using bovine serum albumin as a standard [32]. A volume of 5 μL of each sample and standard was first added to each well then 250 μL of Ready-To-Use Coomassie Blue G-250 Based Reagent (Thermo Scientific, Waltham, MA, USA; Prod no. 23200, lot no. UE285518) was added. The microplate was put on the shaker for 30 s and then incubated for 10 min at RT. Absorbance was read at 570 nm using a microplate reader.

### 2.9. Assessment of Oxidative Stress Markers

The malondialdehyde (MDA) level was estimated using the thiobarbituric acid method as previously described [33]. Liver glutathione was estimated using a colorimetric kit (Biodiagnostics, Egypt; catalog no. GR 25 11) according to the method of Beutler et al. [34]. Superoxide dismutase enzyme (SOD) activity was determined using a colorimetric assay kit (Sigma-Aldrich, USA; catalog no. CS0009, lot no. 0000143658), which is based on the method of McCord and Fridovich (1969) [35].

### 2.10. Enzyme-Linked Immunosorbent Assay (ELISA)

Tumor necrosis factor alpha (TNFα) and tissue inhibitor matrix metalloproteinases 1 (TIMP-1) were measured using an ELISA kit (TNFα, Inova, China; catalog no. In-Ra1344, lot no. 202101, 202201), (TIMP-1, MilliporeSigma, Burlington, MA, USA; catalog no. RAB0471, lot no. 0930I708, 0914I708).

### 2.11. RT PCR

Sections from the liver tissue stored in RNAlater solution were homogenized in lysis buffer with Qiagen TissueLyser LT, and the homogenate was processed for RNA extraction using RNeasy mini kit (Qiagen, Germany; catalog no. 74104, lot no. 157052104). For DNA digestion, 80 μL of DNase I (Genetix Biotech, Asia; catalog no. PGM052, lot no. 362555) was added to each column and incubation was carried out at room temperature for 15 min. For quality and yield determination, the ratio of absorbance at 260 nm and 280 nm was assessed using a NanoDrop™ Spectrophotometer. To confirm RNA integrity, agarose gel electrophoresis was performed according to Lee, Pei Yun et al. (2012) with some minor modifications; RNA samples were run on agarose gel (1% [*w*/*v*] agarose) with ethidium bromide (0.1 μg mL^−1^ EtBr) and subsequently visualized with a UV-illuminator [35]. After quality and yield determination, reverse transcription of 2 µg of total RNA was performed using a High-Capacity cDNA Reverse Transcription Kit. cDNA was used to determine the expression level of the selected genes with RT-PCR. The genes were detected using an SYBR Green PCR kit (Applied Biosystems, Foster City, CA, USA). The primer sequences used are listed in Table 2.

### 2.12. Molecular Docking

Molecular docking studies were carried out using Molecular Operating Environment (MOE 2014.0901) software, Chemical Computing Group, Montreal, Canada, following the docking protocol [36]. The X-ray crystal structure of the TGF-β (PDB ID: 1VJY) active site was downloaded from the RCSB Protein Date Bank website [37]. The validity of the used docking protocol was confirmed when the root-mean-square deviation (RMSD) score was less than 1.5 or 2 Å [38].

### 2.13. Statistical Analysis

The differences between any two groups were analyzed using Student’s *t*-test, and the statistical significance among multiple groups was tested with One-Way ANOVA followed by Tukey’s multiple comparisons test. Data were analyzed using GraphPad Prism version 9.00, GraphPad, Software, San Diego, CA, USA.

## 3. Results

### 3.1. D1-3 In Vitro COX-1/COX-2 Inhibition Ability with 100% Cell Viability

Because the newly synthesized compounds are structurally related to celecoxib, D1-3 were tested for their selective COX-2 inhibition ability, and to anticipate the toxicity and safety of the tested compounds before toxicity testing in experimental animals, an in vitro cytotoxicity assay was carried out. The results showed that the therapeutic IC50 of the tested compounds was in a narrow range from 0.089 to 0.135 µM with good selectivity towards COX-2 compared to COX-1 yet less potent than celecoxib. The results also revealed that upon treatment with up to 10× the therapeutic IC50 on HepG2, no cytotoxicity happened with 100% cell viability. The safety index (SI) of the three compounds was very high as the difference between the cytotoxicity IC50 and Therapeutic IC50 was huge (Table 3).

### 3.2. In Vivo Toxicity Study

#### 3.2.1. Effect of Celecoxib-Based Fused Ring Derivatives on Rats’ Vitals and Body Weight

**Figure 1:**
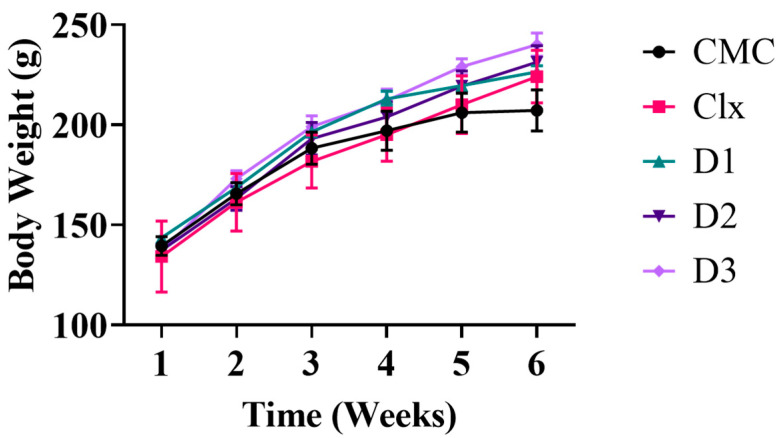
Male albino rats’ body weight through the six weeks of the experiment. Data are represented as mean  ±  SEM. No significant difference was obvious between the groups; *n* = 6.

The toxicity responses resulting from animals can be decisive in judging the safety of chemical compounds if they are found to have potential pharmacological effects. In our study, all rats were observed daily after treatment by compounds under test for the presence of any signs of toxicity. The body weight of each rat was recorded once weekly for 6 weeks until the end of the experiment. No mortality was observed during the experimental period, and all the treated rats survived the 6 weeks of the experiment. During the observation, no toxic signs were obvious on the rats’ vitals. The compounds did not produce any adverse toxic effect on the body weight changes, such that all animals exhibited a normal increase in body weight without any noticeable difference between both the control and treated groups (Figure 1).

#### 3.2.2. Hepatic and Renal Safety of the Compounds

Biochemical parameters are indicative of the functional status of major organs such as the liver, heart, and kidney, so, we determined plasma hepatic biomarkers (ALT, AST, ALP, total bilirubin, and albumin), lipid biomarkers (triglycerides and total cholesterol levels), and renal biomarker (creatinine). The results showed that both the standard drug (celecoxib) and celecoxib-based fused ring derivatives exhibited the same liver, renal, and lipid profile as that of the CMC group confirming the hepatic and renal safety of celecoxib-based fused ring derivatives (Table 4).

#### 3.2.3. D1-3 Preserved Normal Cellular Architecture of the Liver

**Figure 2:**
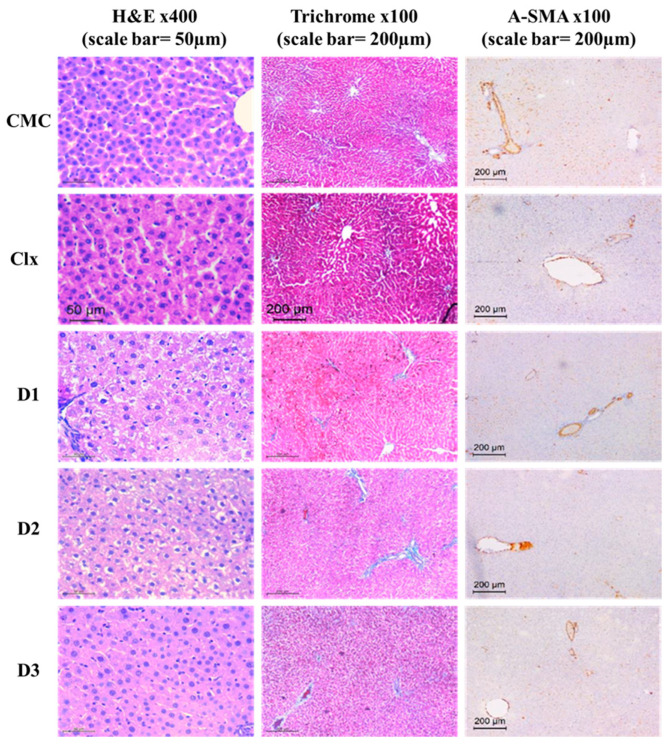
Effect of celecoxib and its fused ring derivatives on liver histology. Celecoxi-based fused ring derivatives showed no pathological effect on liver tissue as represented using hematoxylin and eosin, Masson trichrome, and smooth muscle actin (SMA) monoclonal primary antibody stains.

Histological and immunohistochemistry analyses were performed to further exclude any cellular alteration in the structure of the liver. In this study, evaluation of liver architecture and necrosis using different staining protocols (Hematoxylin and Eosin, Masson trichrome, and smooth muscle actin (SMA) monoclonal primary antibody) revealed normal hepatocytes and liver lobular architecture in CMC-treated rats as well as both standard drug (celecoxib) and celecoxib-based fused ring derivatives-treated rats. Hepatocytes were radiating as thin cords from central veins. They were polygonal with eosinophilic cytoplasm and vesicular nuclei. No cholestasis or steatosis was seen. Bile ducts were detected in portal tracts without any evidence of bile ductular injury or proliferation in the aforementioned groups, substantiating no toxicity on the liver level. Portal tracts were of average size with no evidence of fibrosis in the trichrome-stained sections. The α-SMA positive area constituted only 1.5–4.1% in those groups with no significant difference with the control group (Figure 2).

### 3.3. Therapeutic Model Treated after Induction of Fibrosis

#### 3.3.1. D1-3 Suppressed the Severity of CCl_4_-Induced Fibrosis

**Figure 3:**
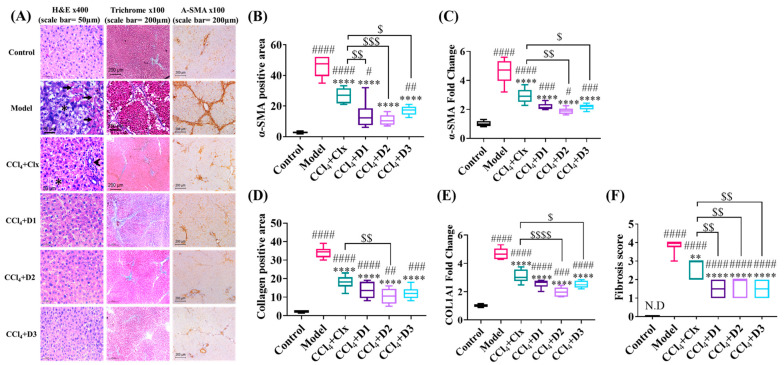
(**A**) Microscopic analysis of liver tissue. The model group showed marked pathological changes in the liver, as shown with hematoxylin and eosin. Multiple councilman bodies are seen (arrows) as well as feathery degeneration of hepatocytes (*). Masson trichrome staining showed nodular liver architecture with thick fibrous septea stained blue. Large areas are stained brown with α-SMA immune staining. Residual inflammation (arrowhead) and feathery degeneration (*) are still seen in the Clx-treated group, while the rest of the derivatives showed variable degrees of restoration of hepatic architecture. (**B**) α-SMA-positive area (%). (**C**) Hepatic mRNA expression of α-SMA (fold change). (**D**) Collagen-positive area (%). (**E**) Hepatic mRNA expression of COL1A1 (fold change). (**F**) Fibrosis score (0–4). Data shown are expressed from minimum to maximum as box and whiskers; *n* = 6. # *p* < 0.05, ## *p* < 0.01, ### *p* < 0.001, and #### *p* < 0.0001 compared to control. ** *p* < 0.01 and **** *p* < 0.0001 compared to model. $ *p* < 0.05, $$ *p* < 0.01, $$$ *p* < 0.001, and $$$$ *p* < 0.0001 compared to CCl_4_ + Clx.

In fact, the regression of fibrosis is characterized by the decline in fibrosis score, collagen, and α-SMA. In our study, CCl_4_ induced evident hepatic damage with a severe fibrotic response that was confirmed by the total loss of normal hepatic architecture, which was replaced by nodules of regenerating hepatocytes separated by thick fibrous septea. Hepatocytes showed feathery degeneration, ballooning, and numerous councilman bodies. Mild to moderate inflammatory infiltrate was seen in fibrous bands and within hepatic lobules. The Masson trichrome-stained sections highlighted the thick fibrotic septea which were bridging from the central veins to portal tracts forming a nodular architecture. Meanwhile, collagen- and α-SMA-positive areas constitute 30 to 39% and 35 to 52%, respectively. After treatment with the derivatives under this experiment, the liver sections showed different degrees of restoration of normal architecture, a decline in the fibrotic score, and a decrease in collagen- and α-SMA-positive areas. This improvement was confirmed by the decreased mRNA expression of α-SMA and collagen type 1 (COL1A1) and was statistically significant in comparison to the CCl_4_ model group. The enhancement exerted by the derivatives was better than that of the Clx-treated group; however, the best effect was exerted by D2 (Figure 3).

#### 3.3.2. Antioxidant Properties of D1-3 in Reducing Hepatic Fibrosis

**Figure 4:**
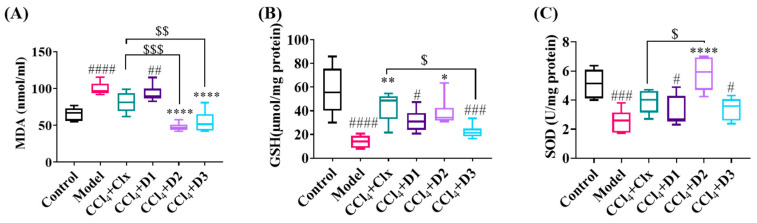
Oxidative stress markers in liver tissue homogenate (**A**) MDA content (nmol/mL), (**B**) reduced GSH (μmol/mg protein), and (**C**) SOD (U/mg protein). Data shown are expressed from minimum to maximum as box and whiskers; *n* = 6. # *p* < 0.05, ## *p* < 0.01, ### *p* < 0.001, and #### *p* < 0.0001 compared to control. * *p* < 0.05, ** *p* < 0.01, and **** *p* < 0.0001 compared to model. $ *p* < 0.05, $$ *p* < 0.01, $$$ *p* < 0.001 compared to CCl_4_ + Clx.

As oxidative stress is one of the pathogenic mechanisms associated with the development and accumulation of fibrosis, we examined the antioxidant capability of the derivatives. In our study, a significant increase in the MDA level happened in the model group compared to the healthy control group that was accompanied with a depletion in reduced GSH and SOD level indicating high oxidative stress and lipid peroxidation as a mechanism of tissue damage. Treatment with D1 showed no significance difference to Clx treatment, while D3 was significantly better that Clx in MDA, equal to it in SOD, and worse in GSH. D2 showed the best enhancement in redox status (Figure 4).

#### 3.3.3. D1-3 Enhanced Liver Enzymes Balance after CCl_4_-Induced Fibrosis

**Figure 5:**
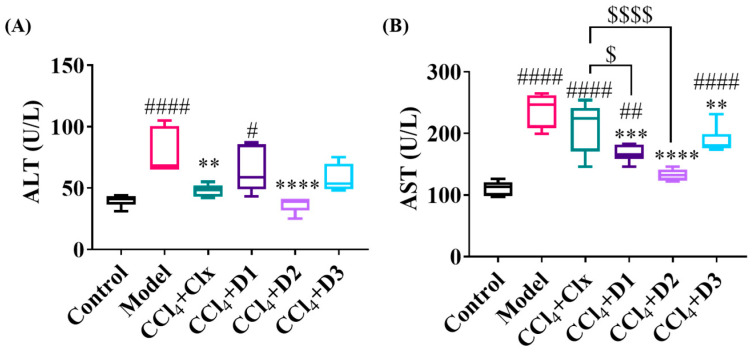
Level of plasma hepatic biomarkers (**A**) ALT (U/L) and (**B**) AST (U/L) in plasma of rats after induction of CCl_4_ and subsequent treatment with either Clx or its derivatives. Data shown are expressed from minimum to maximum as box and whiskers; *n* = 6. # *p* < 0.05, ## *p* < 0.01, #### *p* < 0.0001 compared to control. ** *p* < 0.01, *** *p* < 0.001, **** *p* < 0.0001 compared to CCl_4_ model. $ *p* < 0.05, $$$$ *p* < 0.0001 compared to CCl_4_ + Clx.

Considering the above results, we estimated some hepatic function tests. Estimation of hepatic biomarkers showed that the plasma ALT and AST in the CCl_4_ + CMC-treated rats (model group) were markedly elevated, indicating hepatocellular disease. A recovery of liver function started by a restoration in the increased enzyme levels, as previously mentioned, which were more significant after treating with D2 than treating with Clx, D1, and D3. (Figure 5).

#### 3.3.4. D1-3 Extracellular Matrix Protein Remodeling Capability

**Figure 6:**
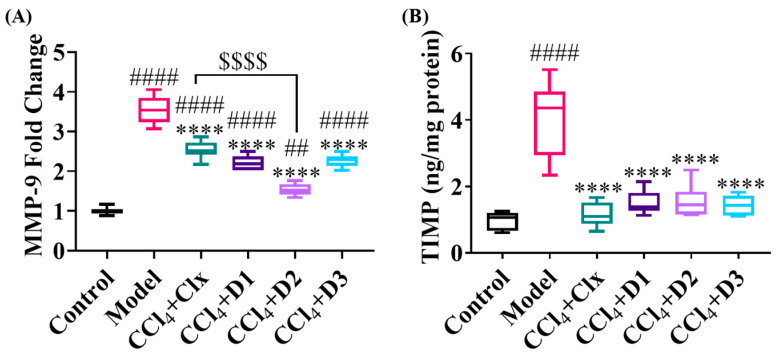
Effect of treatment with various derivatives after CCl_4_ fibrosis induction on ECM remodeling. (**A**) Hepatic mRNA expression of MMP-9 (fold change) and (**B**) hepatic TIMP-1 protein expression level (ng/mg protein). Data shown are expressed from minimum to maximum as box and whiskers; *n* = 6. ## *p* < 0.01, and #### *p* < 0.0001 compared to control. **** *p* < 0.0001 compared to model. $$$$ *p* < 0.0001 compared to CCl_4_ + Clx.

To maintain homeostasis and avoid extra collagen deposition, careful management of ECM remodeling is required. In our study, measurement of MMP-9 revealed significant gene expression in the model group that decreased potentially after treatment with clx and the three drugs but without reaching the control group value. Only D2 showed a more significant reduction, reaching a low MMP-9 level near that of the control group, and was more significantly effective than Clx. Measurement of TIMP-1 showed a significant increase in the model group compared to the control group. Treatment with clx and the three drugs showed a substantial decrease in the TIMP-1 protein level reaching control levels and confirming antifibrotic properties (Figure 6).

#### 3.3.5. D1-3 Reduced Proinflammatory and Profibrogenic Cytokines in Induced Liver Injury

**Figure 7:**
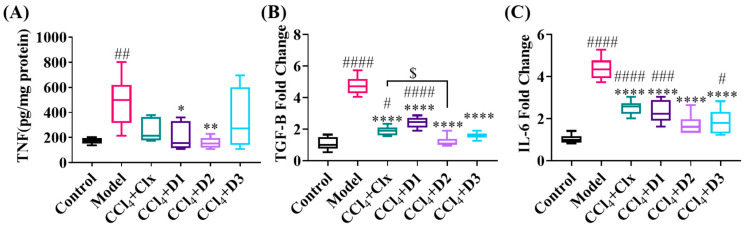
Proinflammatory and profibrogenic cytokines level of expression through fibrosis and after treatment. (**A**) Hepatic TNF-α protein expression level (pg/mg protein), (**B**) hepatic mRNA expression of TGF-β (fold change), and (**C**) hepatic mRNA expression of IL-6 (fold change). Data shown are expressed from minimum to maximum as box and whiskers; *n* = 6. # *p* < 0.05, ## *p* < 0.01, ### *p* < 0.001, and #### *p* < 0.0001 compared to control. * *p* < 0.05, ** *p* < 0.01, and **** *p* < 0.0001 compared to model. $ *p* < 0.05 compared to CCl_4_ + Clx.

As inflammation is one of the major etiological agents leading to liver fibrosis, we detected the severity of inflammation using measurements of TNF-α protein expression level and TGF-β and IL-6 gene expression levels. Measurement of the previously mentioned cytokines showed a significant increase in the fibrosed model that decreased considerably reaching the control level upon treatment with D2, noting that only treatment with D2 was more significant than celecoxib in reducing TGF-β. Clx and the three drugs reduced the increased levels of the previously mentioned cytokines but not as potently as D2 (Figure 7).

### 3.4. In Silico Prediction of D1-3 Possible Mechanism of Action

**Figure 8:**
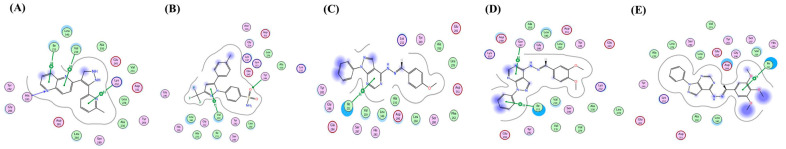
Engagement of Clx and D1-3 in some binding patterns with the TGF-β type 1 receptor active site such as those exhibited by the co-crystallized ligand. (**A**) The co-crystallized ligand, (**B**) Clx, (**C**) D1, (**D**) D2, and (**E**) D3.

Inspired by the promising antifibrotic activities exhibited by compounds D1-3, an in silico molecular docking was carried out to predict their binding mode and molecular interactions with the active site of the TGF-β type 1 receptor as a possible target. Re-docking of the co-crystallized ligand in the TGF-β type 1 receptor active site validated the docking protocol with an RMSD of 0.8089 Å and with a binding energy score of −8.1393 kcal/mol. Molecular modeling revealed that the three investigated compounds D1-3 were satisfactorily located onto the binding site of the TGF-β type 1 receptor active site showing a binding affinity range (−6.53 to −7.50 Kcal/mol) related to that of co-crystallized ligand (−8.13 Kcal/mol) that formed three π–H interactions with Ile211, Val219, and Lys232 and an extra H-bond with Asp351 as the H-bond donor. As shown in Figure 8, these compounds were engaged in several molecular interactions such as those displayed by the co-crystallized ligand. Referring to the docking results of the compounds, the active site of TGF-β type 1 receptor, clx formed one π–H interaction with Val219 and a H-bond with Tyr249 as the H-bond donor with a binding energy of −6.8 kcal/mol, D1 formed one π–H interaction with Gly212 with a binding energy of −6.5 Kcal/mol, D2 formed two π–H interactions with Ile211 and Ser87 with a binding energy of −7.5 Kcal/mol, and D3 formed one π–H interaction with Ile211 with a binding energy of −7.3 Kcal/mol.

## 4. Discussion

A key part of drug discovery and assessment is the safety index (SI) of drug candidates, which is a quantitative ratio of their safety (Cytotoxicity IC50) to their efficacy (in vitro pharmacological COX-2 inhibition IC50). In order for a drug to possess a good safety profile, it should have a high SI [39]. In order to determine the SI of the novel drugs, the in vitro COX-1/COX-2 inhibition ability was estimated as COX-2 can promote the synthesis of prostaglandins and initiate inflammation, which is a potent inducer of hepatic fibrosis [40]. The tested compounds showed selective inhibition to COX-2 with less inhibition potency than celecoxib, which indicates possible anti-inflammatory properties with low gastrointestinal and cardiovascular side effects. This is maybe due to the fact that less inhibition potency to COX-2 can decrease cardiovascular toxicity [41], and the more selective the COX-2 inhibition, the fewer gastrointestinal side-effects would be expected [42]. In order to measure the cytotoxicity (IC50), MTT was conducted to determine cell viability, proliferation, and metabolic activity [43]. Our results showed good SI, confirming that the compounds have a relatively safe in vitro safety profile.

Before the evaluation of the antifibrotic activity of the developed celecoxib analogs, and to further confirm their safety, their toxicity was studied in vivo on normal rats. Rats treated with the compounds under investigation were compared to the control groups treated with CMC. CMC was previously reported as a safe pharmaceutical and food additive [44,45]. Although a high dose (2000 mg/kg) of CMC can induce obvious adverse effects, a low dose (50 mg/kg) of CMC has negligible effects [46]. In our study, 12 mg/kg of CMC could be used safely and considered as normal control rats. We first examined the behavioral and physical appearance features as they are indicators of toxicity [47]. Secondly, we monitored the body weight of the rats in a weekly manner because body weight is an indicative parameter of the animal’s state. The dose determination of tested substances in toxicity studies basically depends on body weight [48]. Thirdly, we checked hepatic and renal enzymes as they can be considered cell damage markers and are commonly used in toxicological studies [49]. Finally, as the liver is the main susceptible organ to the toxic effects of chemicals and chemical interactions because of its fundamental anatomy, capability of xenobiotics clearance from the blood, and high metabolic potential [50], we carried out a liver histopathological examination to exclude any pathological disease [51]. The comparison of the compounds under the investigation-treated rats with the control group (CMC) in all the previously mentioned parameters did not show any significant toxicity. The low adverse effects result obtained from the in vivo toxicity study experiment opens the field for further future application of the studied compounds.

Fibrosis buildup is a dynamic process that results from liver injury. Oxidative stress with increased ROS generation along with depletion in natural non-enzymatic antioxidants such as GSH, and enzymatic antioxidant defenses such as SOD, lead directly to a flaw in the cell redox balance, and indirectly to an increase in matrix production and fibrogenesis [52,53]. Moreover, ROS have been demonstrated to potently induce TNFα [54]. ROS overproduction can initiate lipid peroxidation, which is measured by the formation of a malondialdehyde (MDA) level [55]. Furthermore, ECM remodeling is crucial in tissue regeneration and wound healing [56]. The imbalance in the main enzymes involved in the ECM degradation, such as MMPs and their inhibitors TIMPs is a major aspect concerned with matrix stiffness and consequently the progression of liver fibrosis [57]. One of the MMPs is MMP-9 which helps in liver fibrogenesis regulation and fibrosis resolution [58]. Nevertheless, increased evidence has demonstrated that MMP-9 is a double-edged sword as it indorses fibrogenesis by actuating TGF-β, which stimulates HSC activation [59]. It has been acknowledged that TIMP-1 plays a prevalent role in fibrosis progression by inhibiting the activity of MMPs [60]. In addition, the upregulated release of proinflammatory (TNF-α and IL-6) and profibrogenic (TGF-β) cytokines by Kupffer cells and stimulated HSCs is one of the first manifestations of liver fibrosis [61]. TNF-α plays a vital role in many stages of liver diseases as the degree of fibrosis develops via its mediated chronic inflammation [62]. Although IL-6 historically was known to induce liver inflammation and fibrosis as well as collagen synthesis [63], recent studies revealed that IL-6 is associated with protective functions throughout hepatic fibrogenesis [64]. IL-6 can act as both a pro- and an anti-inflammatory cytokine that may attribute to liver damage through different mechanisms [65]. Interestingly, mice with ablated IL-6 had aggravated hepatocyte damage which led to more severe liver fibrosis [66]. In the case of IL-6, caution should be taken in the treatment of hepatic fibrosis as it prevents fibrogenesis yet increases the risk of HCC occurrence [67,68].

TGF-β contributes to various stages of hepatic-disease progression, from liver inflammation to HCC [69]. When wounding or inflammation takes place, lymphocytes, monocytes/macrophages, and platelets produce TGF-β among the profibrotic mediators, [70,71] which activates HSC to myofibroblasts that are the key source of ECM accumulation and fibrogenesis progression [72]. Interestingly, liver fibrosis with varying degrees of hepatic architectural distortion and collagen deposition are well identified histologically with hematoxylin–eosin sections or with histochemical stains such as Masson’s trichrome [73,74].

The up-regulation of α-SMA is a fundamental step that goes in parallel with HSC activation and liver fibrosis [75]. In fact, the regression of fibrosis is characterized by a decline in the fibrosis score and α-SMA. Additionally, ALT and AST are hepatic-enzyme indicators of liver injury. When damage happens to hepatocytes, ALT and AST were upraised in the bloodstream before the occurrence of clinical signs and symptoms of liver diseases [76,77].

So, to test the anti-fibrotic potential of the compounds, liver fibrosis was firstly induced using CCl_4_ IP injection due to its known classic toxicological mechanism in the induction of liver lesion by multiple biological processes, pathways, and targets [78,79,80], followed by treatment with celecoxib or the compounds.

Unluckily, there is controversy about celecoxib’s effectiveness against induced liver fibrosis in the literature. Although celecoxib (20 mg/kg/day orally) had revealed potentiality in the attenuation of induced liver fibrosis as reported [81,82,83,84], other studies stated that it (15 mg/kg/day orally) had no effect on induced liver fibrosis in rat models [85,86,87] or on mice models when injected subcutaneously (3.6 µL/day) [88]. The different exposure times and methods to the fibrotic agent and the dose and duration of celecoxib might elucidate the contradictory results [14]. The previously mentioned studies are considered preventive models during fibrosis induction to determine celecoxib’s capacity to prevent liver fibrosis, while the current study is considered a therapeutic model treated with celecoxib after the induction of fibrosis to investigate its fibrolytic capability. Furthermore, it is generally acknowledged that the liver fibrogenesis hallmark is the trans-differentiation of resting HSCs into myofibroblast. Many tyrosine kinases (TKs) were found to be significantly expressed in the activated HSCs and during the development of liver fibrosis. Moreover, TKs play a role in angiogenesis through the progression of liver fibrosis [89,90]. It is widely reported that several TK inhibitors exhibit anti-liver fibrotic activity [91,92,93,94]. Hence, we examined for the first time the hepatoprotective effect of three celecoxib structurally related derivatives that were reported as kinase inhibitors having antineoplastic activity [21,95].

The CCl_4_-induced liver fibrosis model was successful as the fibrosis severity and the fibrotic parameters were significantly increased compared to the normal control group; *p* < 0.0001. In line with Chávez et al. (2010) [14] and Ftahy et al. (2013) [15], we found that celecoxib had therapeutic effects against liver fibrosis such that it reduced the CCl_4_-induced high plasma ALT level, fibrotic score, α-SMA positive area, oxidative stress markers, proinflammatory and profibrogenic cytokines, MMP9, and TIMP-1 compared to the CCl_4_ model group; *p* < 0.05. The results also revealed that the therapeutic effect against liver fibrosis of the three celecoxib structurally related derivatives was established more significantly than celecoxib and, in particular, by the dimethoxy derivative (D2). The enhanced fibrolytic effect of the derivatives could be explained due to their structure–activity relationship (SAR) as a literature survey indicated that the inclusion of alkoxy substituents (methoxy, aryloxy and/or methylenedioxy moieties) within the structure could significantly enhance a variety of biological activities due to the expected intensification of the compounds’ lipophilicity [96,97,98,99]. In particular, the impact of the number and position of methoxy substituents on the extent of several bioactivities including the antioxidant and antiproliferative efficiencies was recently reported [100,101].

## 5. Conclusions

The dimethoxy Pyrazolo[3,4-d]pyrimidine derivative could be considered as a safe and promising antifibrotic analog as it exhibited the greatest therapeutic activity against fibrosis in all the tested parameters compared to the reference drug celecoxib and the other two analogs. It showed a significant reduction in CCl_4_-induced elevations in plasma ALT and AST in addition to a reduction in the oxidative stress parameters, an observable decrease in the fibrotic score and α-SMA positive area, and a marked reduction in the protein expression level of TNF-α and TIMP-1 and gene expression level of TGF-β and IL-6. Its antifibrotic effect could be through the TGF-β signaling pathway as proposed by molecular docking; however, additional experiments are needed to further confirm these findings along with the optimum dose and the exact mechanism of action.

## Figures and Tables

**Table 1:** Experimental animal design.

Main Group	Weeks	1	2	3	4	5	6	7	8	9	10	11	12
Toxicity study (*n* = 40)	CMC (*n* = 8)	2.4 mL/kg of CMC (0.5%) in deionized water, orally, daily						-					
	Clx (*n* = 8)	20 mg/kg of celecoxib (8.33 mg/mL) in 0.5%CMC, orally, daily [15,27]						-					
	D1 (*n* = 8)	D1 was given orally in a dose equimolar to celecoxib;18.058 mg/kg/day						-					
	D2 (*n* = 8)	D2 was given orally in a dose equimolar to celecoxib;18.18 mg/kg/day						-					
	D3 (*n* = 8)	D3 was given orally in a dose equimolar to celecoxib;21.2 mg/kg/day						-					
Therapeutic model (*n* = 58)	Control (*n* = 8)	1.2 mL/kg of corn oil, IP, twice a week						CMC was given orally (same dose as CMC group)					
	Model (*n* = 10)	2 mL/kg of CCl_4_ (40%) in corn oil, IP, twice a week [28]						CMC was given orally (same dose as CMC group)					
	CCl_4_ + Clx (*n* = 10)	CCl_4_ was injected (same dose as model group)						Clx was given orally (same dose as Clx group)					
	CCl_4_ + D1 (*n* = 10)	CCl_4_ was injected (same dose as model group)						D1 was given orally (same dose as D1 group)					
	CCl_4_ + D2 (*n* = 10)	CCl_4_ was injected (same dose as model group)						D2 was given orally (same dose as D2 group)					
	CCl_4_ + D3 (*n* = 10)	CCl_4_ was injected (same dose as model group)						D3 was given orally (same dose as D3 group)					

**Table 2:** Primers used for real-time quantitative PCR.

Gene Symbol	Forward Primer Sequence	Reverse Primer Sequence
GAPDH	GTA TTG GGC GCC TGG TCA CC	CGC TCC TGG AAG ATG GTG ATG G
TGF-β	ATC CCT GCG ACC CAC ACA AG	CAA CTG CTT TGG AAG GAC TCG
MMP-9	CAATCCTTGCAATGTGGATG	TAAGGAAGGGGCCCTGTAAT
Il-6	TGA TGG ATG CTT CCA AAC TG	GAG CAT TGG AAG TTG GGG TA
α-SMA	CGA TAG AAC ACG GCA TCA TCA C	GCA TAG CCC TCA TAG ATA GGC A
COL1A1	CAT GTT CAG CTT TGT GGA CCT	GCA GCT GAC TTC AGG GAT GT

GAPDH, glyceraldehyde 3-phosphate dehydrogenase; TGF-β, transforming growth factor beta; MMP-9, matrix metallopeptidase 9; IL-6, interleukin 6; α-SMA, alpha-smooth muscle actin; COL1A1, collagen type 1.

**Table 3:** In vitro COX-1/COX-2 inhibition ability of the tested compounds and their cytotoxicity against hepG2. Values are expressed as mean  ±  SEM; (*n* = 3).

Compound	In Vitro Inhibition IC50 ± SEM (µM)		Selectivity Ratio COX-1/COX-2	Cytotoxicity IC50 ± SEM (µM)	Safety Index (SI) *
	COX-1	COX_2			
D1	7.5 ± 0.09	0.135 ± 0.0004	55.5	370.7 ± 0.089	2745.9
D2	8.5 ± 0.04	0.103 ± 0.028	82.5	344.95 ± 0.30	3349.0
D3	11.5 ± 0.05	0.089 ± 0.0004	129.2	356.5 ± 2.9	4042.7
Celecoxib	14.5 ± 0.047	0.046 ± 0.001	312.9	144.63 ± 1.08	3144.1
Indomethacin	0.099 ± 0.0004	0.079 ± 0.0004	1.25		

* Safety index is the ratio of Cytotoxicity IC50 to in vitro COX-2 Inhibition IC50.

**Table 4:** Biochemical parameters of in vivo toxicity study group.

	Vehicle Control (CMC)	Clx	D1	D2	D3
Cholesterol (mg/dL)	64.5 ± 4.98	52.5 ± 2.21	71.66 ± 7	67 ± 6.47	62.33 ± 1.68
Triglycerides (mg/dL)	49.16 ± 9.18	53 ± 7.11	67 ± 5.42	61.83 ± 7.11	64 ± 8.01
ALT (U/L)	37.33 ± 2.99	42 ± 3.33	49.66 ± 4.99	39.66 ± 2.13	37.83 ± 2.45
AST (U/L)	156.16 ± 9.68	147.5 ± 18.65	139.5 ± 7.78	156.16 ± 9.68	147.33 ± 9.81
ALP (U/L)	133.5 ± 6.67	138.66 ± 13.40	159.5 ± 7.48	154.16 ± 10.92	123.16 ± 4.12
Total Bilirubin (mg/dL)	0.081 ± 0.01	0.084 ± 0.006	0.073 ± 0.004	0.071 ± 0.01	0.076 ± 0.006
Albumin (g/dL)	3.78 ± 0.09	3.73 ± 0.06	3.9 ± 0.12	3.78 ± 0.12	3.86 ± 0.12
Creatinine (mg/dL)	0.40 ± 0.05	0.35 ± 0.01	0.36 ± 0.03	0.37 ± 0.01	0.42 ± 0.02

Values are expressed as a mean  ±  SEM; (*n* = 6), No significant difference was obvious between the groups.

## Data Availability

Data are contained within the article.
